# A deep learning model for automated identification of age-related macular degeneration atrophy

**DOI:** 10.1007/s00417-026-07205-4

**Published:** 2026-05-09

**Authors:** Oren Avram, Yahel Shwartz, Adi Green, Rivka Bloom, Giulia Corradetti, Anthony Wu, Zeyuan Johnson Chen, Tal Eshkoly Lior, Berkin Durmus, Akos Rudas, Ravi Pal, Nadav Rakocz, Ceren Soylu, Mai Alhelaly, Giacomo Boscia, Charles C. Wykoff, Maxime Cannesson, Srinivas R. Sadda, Jaime Levy, Eran Halperin, Jeffrey N. Chiang, Itay Chowers, Liran Tiosano

**Affiliations:** 1 Department of Computational Medicine, University of California Los Angeles, Los Angeles, CA USA; 2 Department of Computer Science, University of California Los Angeles, Los Angeles, CA USA; 3 Department of Anesthesiology and Perioperative Medicine, University of California Los Angeles, Los Angeles, CA USA; 4 Optum AI, UnitedHealth Group, Eden Prairie, MN USA; 5 Department of Ophthalmology, Hadassah Medical Organization and Faculty of Medicine, Hebrew University of Jerusalem, Jerusalem, Israel; 6 Doheny Eye Institute, Pasadena, CA USA; 7 Department of Ophthalmology, University of California Los Angeles, Los Angeles, CA USA; 8 Stein Eye Institute, University of California Los Angeles, Los Angeles, CA USA; 9 Office of Health Informatics and Analytics, UCLA Health, Los Angeles, CA USA; 10 Retina Consultants of Texas, Retina Consultants of America, Houston, TX USA; 11 Blanton Eye Institute, Houston Methodist Hospital, Houston, TX USA; 12 Department of Computer Science, New York University, New York, NY USA; 13 Division of Precision Medicine, New York University, New York, NY USA; 14 Department of Neurosurgery, University of California Los Angeles, Los Angeles, CA USA

**Keywords:** Age-related macular degeneration (AMD), CRORA, Optical coherence tomography (OCT), Deep learning, 3D medical imaging, Retinal atrophy

## Abstract

Age-related macular degeneration (AMD), a leading cause of visual impairment and blindness among the elderly, is projected to affect 288 million individuals globally by 2040. Advanced AMD, including complete retinal pigment epithelium and outer retinal atrophy (cRORA), pose significant challenges for diagnosis and monitoring due to the labor-intensive, costly, and variable nature of manual annotation of volumetric optical coherence tomography (OCT) scans. Automating cRORA diagnosis offers the potential to improve annotation consistency and reduce clinical burden, which could facilitate, for example, the evaluation of recently FDA-approved treatments that delay disease progression. In this study, we compiled two large independent cohorts totaling nearly 5,000 3D OCT scans, labeled them for cRORA presence, and developed a deep learning model for cRORA automated detection. The model achieved state-of-the-art performance, with a ROC AUC of 0.97 on internal validation, and demonstrated robust translatability (zero-shot learning) with a ROC AUC of 0.88 on external evaluation. Notably, it exhibited high accuracy for both non-neovascular (non-nv) and neovascular (nv) AMD subgroups (ROC AUC 0.98 and 0.93, respectively), including complex cases with exudation. This model and dataset combination could facilitate clinical research and trial analyses by providing scalable, standardized assessments across non-nv and nv AMD patient subgroups.

**Supplementary Information:**

The online version contains supplementary material available at https://doi.org/10.1007/s00417-026-07205-4.

## Key messages

*****What is known***:**

- Identifying cRORA and related AMD atrophic changes on 3D OCT scans requires expert review and substantial time, which limits large scale research and clinical workflows.
- Existing automated approaches for AMD atrophy detection show variable performance and limited generalizability across datasets and graders.

*****What is new***:**

- This study presents the first evaluation of SLIViT, a state-of-the-art AI model for 3D scans, on a large real-world dataset, tailored for automated cRORA identification across nearly five thousand OCT volumes from two independent cohorts.
- The model achieves high accuracy internally and robust zero shot generalization externally, outperforming benchmark methods while remaining consistent with multiple specialist grading groups.
- The method demonstrates reliable performance across AMD subgroups, defined by neovascular status, including complex exudative and anti VEGF treated cases, supporting practical use in clinical and research settings.
- The final optimized model checkpoint is publicly released to enable further research and retrospective clinical studies, including analyses of atrophy occurrence across datasets and investigations into longitudinal progression (which would require dedicated segmentation/regression endpoints).

**Supplementary Information:**

The online version contains supplementary material available at https://doi.org/10.1007/s00417-026-07205-4.

## Introduction

Age-related macular degeneration (AMD) is a leading cause of irreversible vision loss worldwide, with late-stage disease characterized by retinal atrophy resulting from degeneration of the retinal pigment epithelium and photoreceptors in both non-neovascular (non-nv) [1, 2] and neovascular (nv) AMD [3, 4]. These atrophic changes represent a clinically meaningful endpoint associated with substantial visual morbidity [2, 5].

Spectral-domain optical coherence tomography (OCT) is the reference standard for evaluating AMD-related atrophy and underpins the OCT-based definitions established by the Classification of Atrophy Meetings (CAM) group [2, 6]. These consensus definitions provide standardized criteria for identifying AMD-related atrophic changes across clinical and research settings.

**Fig. 1:**
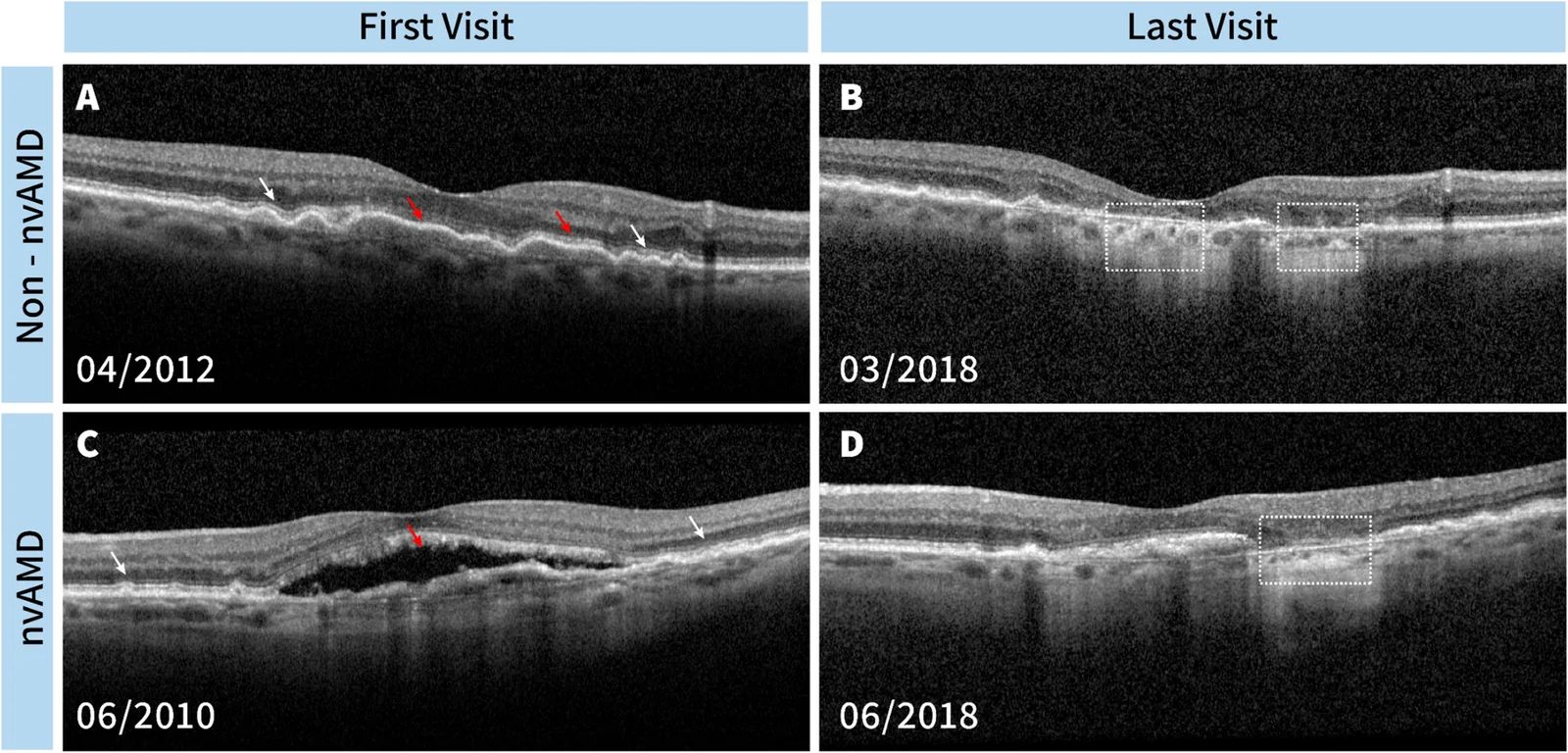
2D OCT B-scan examples of non-nvAMD and nvAMD cRORA-developed patients. An OCT volume’s central slice from scans acquired in two different visits of a non-nvAMD patient (top) and an nvAMD patient (bottom) without (left) and with (right) cRORA. (**A**) Initial visit showing several drusen (white arrows) and PED (red arrows). (**B**) A follow-up scan a few years later reveals two cRORA regions (white boxes) characterized by hypertransmission into the choroid, retinal pigment epithelial disruption > 250 μm, and photoreceptor degeneration, including disruption of the ellipsoid zone and external limiting membrane, thinning of the outer nuclear layer, and subsidence of the outer plexiform layer and inner nuclear layer. (**C**) Initial visit displaying several drusen and subretinal fluid in the foveal area (red arrow), indicative of MNV. (**D**) A follow-up scan a few years later shows absence of fluid following anti-VEGF injections. cRORA is observed in the same region where the MNV was previously identified.

Complete retinal pigment epithelium and outer retinal atrophy (cRORA; Fig. 1) represents an advanced stage of AMD-related atrophy [5] and is frequently first documented when already present. Identifying cRORA across volumetric OCT scans is time-intensive due to the need to review numerous B-scans, particularly in the presence of neovascular changes that can obscure atrophic features. Manual annotation therefore requires specialized expertise, is labor-intensive, and is subject to Inter-clinician variability, contributing to inconsistencies across clinical practice and trials [7].

Automated AI-based annotation of OCT volumes can reduce clinical burden and improve labeling consistency at scale, enabling more efficient cohort construction and longitudinal monitoring across institutions [8]. These advantages are particularly relevant for standardized assessment of AMD-related atrophy in large, heterogeneous real-world datasets [7]. Prior AI studies have shown promising performance for detecting [9] and segmenting [10–12] macular atrophy on OCT. However, many are limited by modest dataset sizes and restricted case-mix, often focusing on non-nvAMD while excluding nvAMD. For example, Chiang, et al., trained exclusively on non-nvAMD patients and, while performing well in that setting, showed a marked performance decline when evaluated on a more diverse external OCT cohort [9]. This highlights the need for robust and generalizable models that perform reliably across heterogeneous populations.

In this study, we have compiled an exceptionally-large multi-site cohort composed of two independent datasets (collected and annotated in different continents) with nearly 5,000 3D OCT scans from more than 2,500 patients. For comparison, our cohort was four to 10 times larger than the number of scans included in previous 3D OCT-based AI studies [7]. We annotated all its volumes with respect to cRORA and used it to develop and evaluate a state-of-the-art deep learning model for fully automated cRORA identification. We showcased its applicability and translatability by evaluating it both internally on our larger dataset (3,883 volumes), and externally (zero-shot learning), on our second dataset (964 volumes). We also conducted differential analysis and demonstrated our trained model’s robustness in cRORA classification in non-nvAMD and nvAMD. Lastly, the final optimized model checkpoint was made available to the community for downstream clinical research and research applications.

## Results

### A specialized model for accurate cRORA classification

Several deep-learning models that allow 3D OCT analysis exist [9, 13–15]. In this work, we used a recently developed model named SLIViT [13] that obtained state-of-the-art performance in a wide variety of 3D medical imaging tasks, including 3D OCT analysis.

For this study, we compiled the Hadassah Dataset (see “Datasets” section). In brief, the dataset consists of nearly 4,000 OCT volumes that were randomly sampled from a larger collection accumulated over more than a decade as part of a longitudinal study on AMD progression in an elderly population. Each OCT volume was labeled for the presence of cRORA by three certified graders from the Hadassah Reading Center.

We conducted an extensive hyperparameter tuning search on a small subset of the data to tailor and specialize the architecture for our task (see “Model Development and Analysis” section). We then leveraged our dataset and the optimal hyperparameter configuration we observed, and trained this promising architecture to classify cRORA. The final model’s checkpoint, trained on all OCT volumes from the Hadassah dataset (and evaluated on an independent dataset) is available for further research via our project’s repository (see “Data and Code Availability” section).

### Model evaluation

**Fig. 2:**
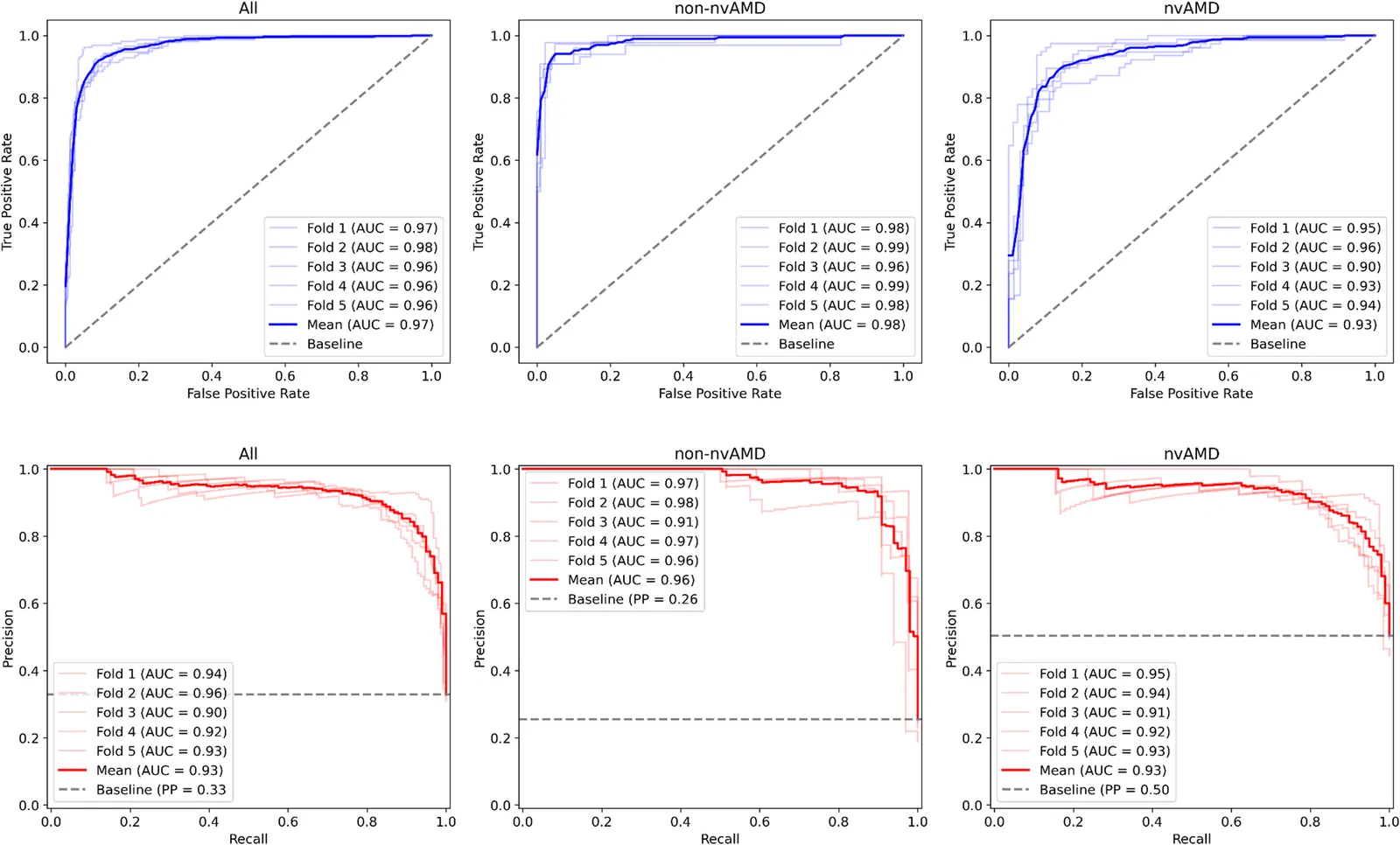
Performance evaluation for cRORA diagnosis (Hadassah dataset). Shown is our trained model’s performance on an internal validation set, measured by ROC AUC (blue) and precision-recall (PR) AUC (red). Each column displays the performance of a different patient subgroup. In each panel, the faint lines represent the performance of individual cross-validation folds, while the bold line represents their average (see also Table 1). The dashed lines indicate the expected performance of a random classifier (0.5 for ROC AUC and the positive-label prevalence (PP) for PR AUC).

We first aimed to evaluate our trained model’s performance on the Hadassah dataset. To do so, we split the dataset at patient level (that is, each patient exclusively resides either in the train, validation, or evaluation set) into train (70%), validation (15%), and test (15%) sets, ending up with sets containing 2,718, 578, and 587 3D OCT volumes, respectively, scanned from 1,071, 230, and 230 patients. This ensured that all OCT volumes from a given individual, including both eyes and all available visits, were confined to a single data split, thereby preventing data leakage and inflation of performance due to correlated samples. In this experiment, we trained our model roughly according to the protocol described in the original SLIViT paper (see Methods for exact details). We measured the receiver operating characteristic (ROC) area under the curve (AUC) and the average precision (AP; also known as the precision-recall AUC). While ROC AUC is a standard metric for binary classification tasks, AP is particularly valuable for imbalanced data (as in our case), as it captures the trade-off between positive predictive value and sensitivity, making it a critical indicator of practical performance. Notably, the volumes in this real-world clinical dataset varied in their characteristics (e.g., number of 2D B-scans; see “Model Development and Analysis” section). Yet, as detailed in Table 1 and illustrated in the leftmost panels of Fig. 2, our optimized model achieved high performance, with 0.97 ROC AUC and 0.93 AP (see also Appendix Table 1 for sensitivity and specificity). We also evaluated other relevant benchmarks. The first was SLIVER-net, a deep-learning method optimized for OCT biomarker classification. A second method was a standard 3D ResNet, largely considered a solid baseline and evidently, very popular not only for 3D OCT [13, 14] but also other volumetric medical imaging modalities [16–18]. Both methods underperformed compared to our specialized model. SLIVER-net achieved 0.91 ROC AUC and 0.8 AP, while 3D ResNet manifested lower performance with 0.72 ROC AUC and 0.51 AP. Despite being less suited to our (volume-level labeled) dataset, we also attempted to train and evaluate a recently developed 2D-ResNet-based model [9]. Nevertheless, its performance was not better than those of the least performative method (see Benchmarking section).

**Table 1:** Performance evaluation for cRORA diagnosis (Hadassah dataset).

Subgroup	Metric	SLIViT	SLIVER-net	3D ResNet
All (n=587; PP =. 35)	ROC AUC	.97	.91	.72
	AP	.93	.80	.51
non-nvAMD (n=235; PP =. 3)	ROC AUC	.98	.94	.76
	AP	.96	.81	.49
nvAMD (n=187; PP =. 53)	ROC AUC	.93	.80	.65
	AP	.93	.79	.63

Shown are the five-fold cross-validation average performance values underlying Fig. 2. The top-performing method for each metric and subgroup is highlighted in bold. The test size (n) and the cRORA positive prevalence (PP) for each subgroup are provided in parentheses.

In addition, as mentioned earlier, there is a considerable clinical importance to diagnose cRORA in different AMD subgroups defined by neovascular status. Therefore, we conducted a differential performance analysis where we evaluated (using the same trained model) subsets of our test set that were AMD homogeneous with respect to neovascular status (Fig. 2; Table 1). Out of the 587 test volumes, 235 were in the non-neovascular (non-nv) phase and 187 were in the neovascular (nv) phase. Interestingly, differentially analyzing these patient groups was not detrimental to performance. For non-nvAMD patients, our model achieved 0.98 ROC AUC and 0.96 AP significantly outperforming the second-best method, SLIVER-net, with 0.94 ROC AUC and 0.81 AP. For nvAMD patients, our model obtained 0.93 for both ROC AUC and AP, significantly outperforming SLIVER-net with 0.8 ROC AUC and 0.79 AP.

### Model translatability

**Fig. 3:**
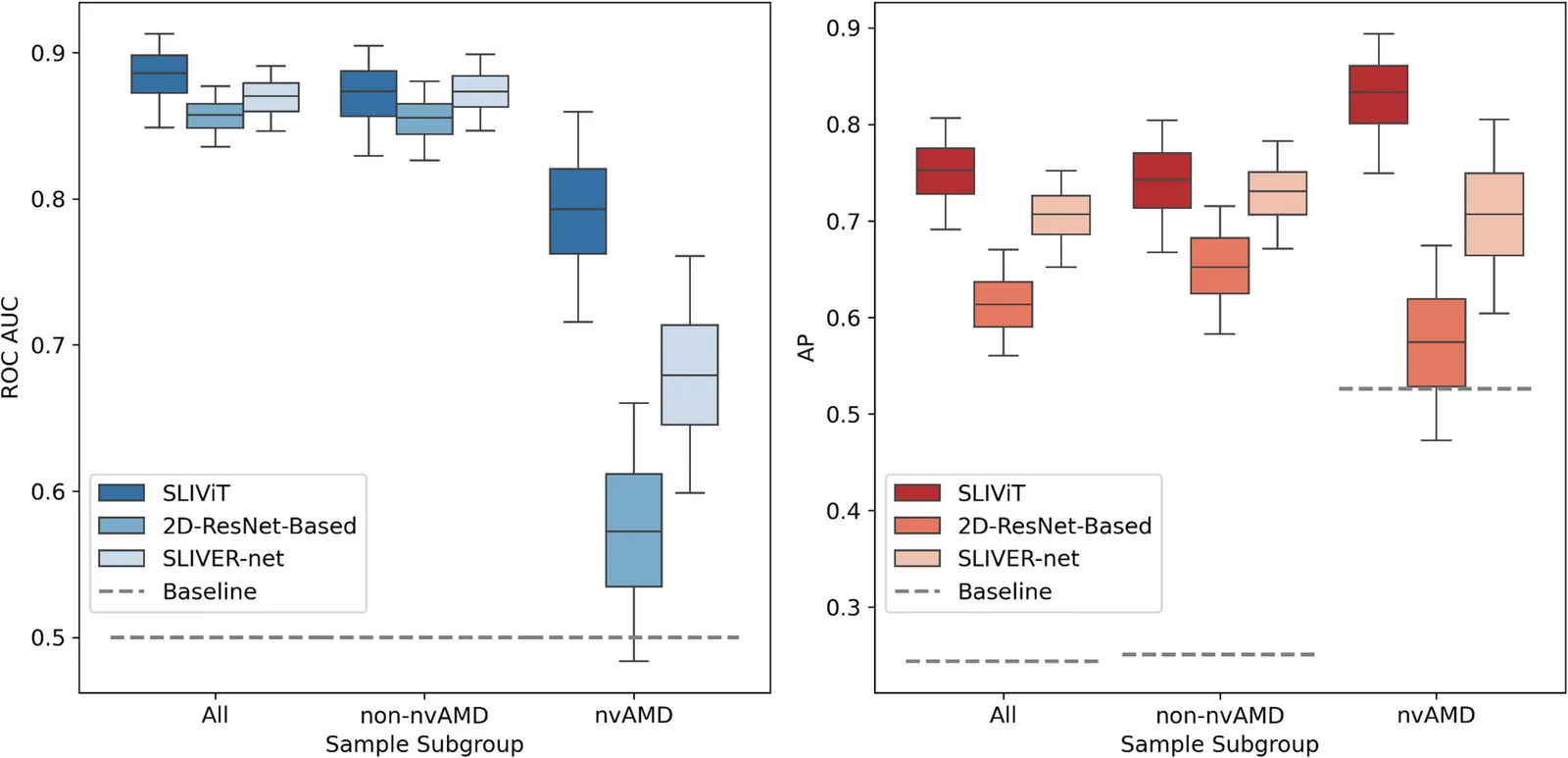
Translatability evaluation for cRORA diagnosis (Houston dataset). Shown are the performance scores in a zero-shot evaluation in three patient subgroups (all patients, non-nvAMD patients, and nvAMD patients) on a set collected from a different clinical institute (see also Table 2). The dashed lines represent the expected performance of a random classifier (which is .5 for ROC AUC and the positive-label prevalence for AP). Box plot whiskers represent a 90% CI.

To assess our model’s application generalizability we next explored its zero-shot performance on an independent dataset, which differed in terms of patients, scanning facility, and the certification of the specialists who annotated the data. To this end, we compiled the Houston Dataset (see Methods) consisting of 964 OCT B-scan volumes. We then trained our model on the entire 3,883 OCT volumes of the Hadassah dataset and evaluated it on the Houston dataset (Fig. 3; Table 2). In this external validation analysis, we obtained 0.88 ROC AUC (CI [0.85, 0.91]) and 0.75 AP (CI [0.69, 0.81]). When repeating the differential performance analysis for the different AMD subgroups, we observed 0.87 ROC AUC (CI [0.83, 0.91]) and 0.74 AP (CI [0.67, 0.8]) for non-nvAMD patients, and for nvAMD patients, obtained 0.79 ROC AUC (CI [0.72, 0.86]) and 0.83 AP (CI [0.75, 0.89]). Although these numbers might initially suggest a notable performance reduction, several sources of domain shift may contribute to this difference, including Inter-clinician variability [19] as discussed later on in the “Inter-clinician variability” section.

**Table 2:** Translatability evaluation for cRORA diagnosis (Houston dataset).

Subgroup	Metric	SLIViT	SLIVER-net	2D ResNet-based
All (n=964; PP = .24)	ROC AUC	.88 [.85, .91]	.87 [.85, .89]	.86 [.84, .88]
	AP	.75 [.69, .81]	.71 [.65, .75]	.61 [.56, .67]
non-nvAMD (n=666; PP = .25)	ROC AUC	.87 [.83, .90]	.87 [.85, .90]	.85 [.83, .88]
	AP	.74 [.67, .80]	.73 [.67, .78]	.65 [.58, .72]
nvAMD (n=114; PP =. 53)	ROC AUC	.79 [.72, .86]	.68 [.60, .76]	.57 [.48, .66]
	AP	.83 [.75, .89]	.71 [.60,.81]	.57 [.47, .67]

Shown are the zero-shot performance values underlying Fig. 3. The square brackets next to each performance score represent a 90% CI. The score of the top-performing method in each patient subgroup appears in bold. The test size (n) and the cRORA positive prevalence (PP) for each subgroup are provided in parentheses.

For the sake of comparison, we also evaluated the translatability of a recently developed 2D ResNet-based deep-learning model that was specialized particularly for cRORA diagnosis [9]. This model was trained exclusively on non-nv AMD using slice-level supervision; accordingly, while its evaluation on the non-nv subgroup reflects in-domain performance, its application to the full cohort and to nv AMD represents a zero-shot setting. For the inference analysis, we borrowed the pre-trained checkpoint of the model (instead of training it ourselves). Of note, the annotations of the dataset on which this model was originally trained and the ones of the evaluation dataset were procured by graders with the same clinical certification (Doheny Image Reading Research Laboratory). This poses an additional challenge for our model as it reduces potential Inter-clinician variability between the train and test sets of the 2D-ResNet-based competitor model, and could thus theoretically confer a performance advantage. Nevertheless, our model outperformed this 2D-ResNet-based competitor on the zero-shot evaluation (Fig. 3; Table 2). For example, when we considered the nvAMD subgroup, it outperformed the competitor in both ROC AUC (0.57; CI [0.48, 0.66]) and AP (0.57; CI [0.47, 0.67]). In addition, we evaluated a SLIVER-net model, that was subject to the same training scheme as our model, on the Houston dataset. Again, our trained model was never outperformed in any of the AMD subgroups (in both metrics), further highlighting the strength of this dataset and model combination. 3D ResNet was also evaluated and underperformed all other methods (with 0.64 ROC AUC (CI [0.61, 0.68]) and 0.39 AP (CI [0.34, 0.44]).

### Translatability vs. training set size dynamics

**Fig. 4:**
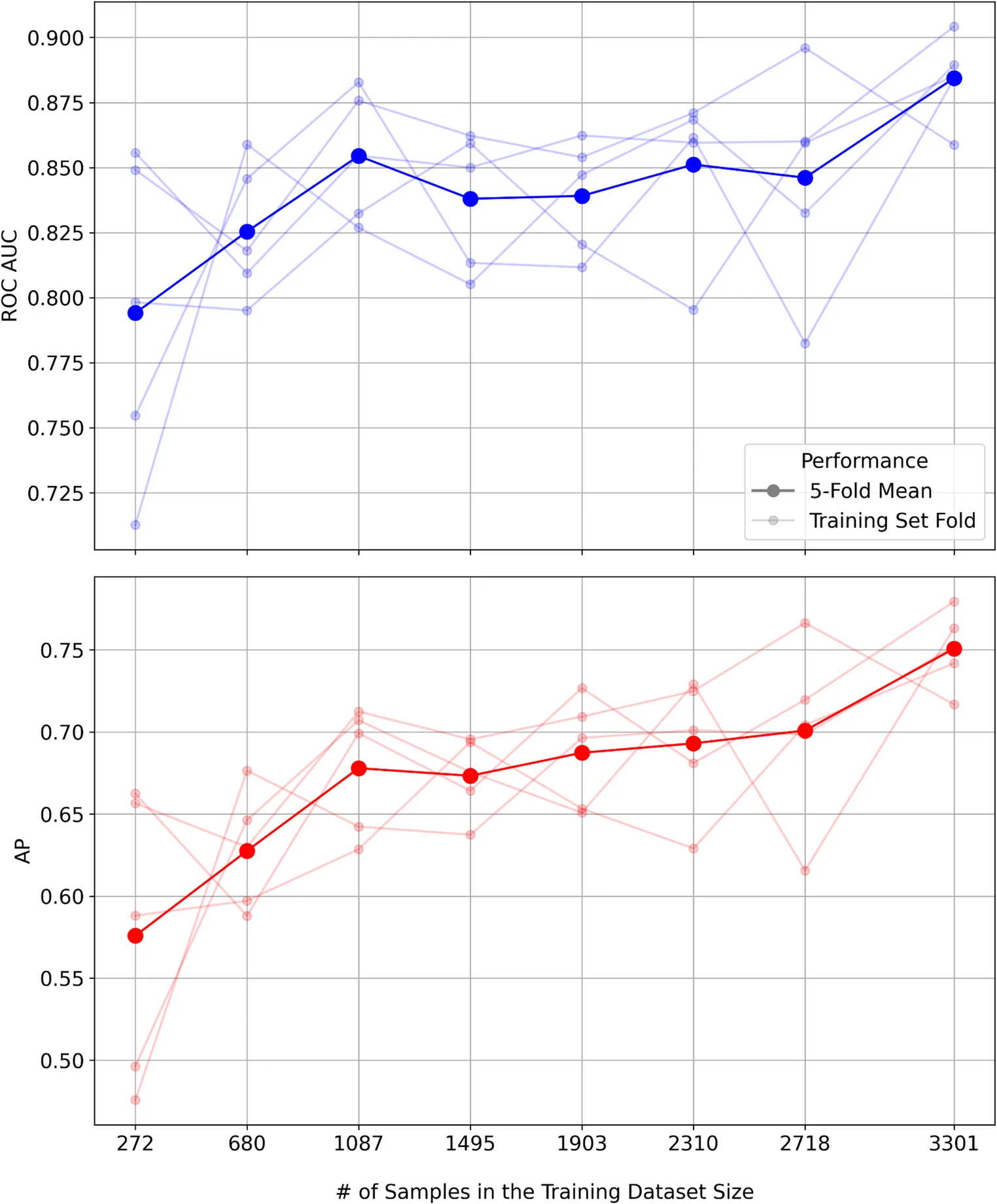
Translatability as a function of training set size. Shown is the model’s overall zero-shot performance when trained on increasing number of samples and tested on an independent dataset. The bold line in each panel represents the five-fold cross-validation average performance, while the faint lines show the performance for individual cross-validation folds.

To highlight the significant contribution of the Hadassah dataset, we investigated how the training set size impacts zero-shot performance on an external dataset (Fig. 4). We fixed 582 volumes (15% of the Hadassah dataset) for model validation, using them after each epoch to monitor improvement. We then randomly sampled size-increasing subsets from the remaining 3,301 samples (85%) and trained our model on each subset. As expected, performance on the external dataset improved as the training set size increased, consistent with previous findings [20]. For instance, training on 1,087 volumes (approximately 25% of the Hadassah dataset), we achieved 0.85 ROC AUC (CI [0.83, 0.88]) and 0.68 AP (CI [0.63, 0.71]). Notably, most 3D OCT AI-based studies typically rely on only dozens to hundreds of volumes [7], representing 25% or less of the Hadassah dataset. Although performance appeared to plateau around this point, further significant improvements were observed when the entire Hadassah dataset was used, underscoring the substantial contribution of its size.

### Inter-clinician variability

To assess the potential for further performance improvement of our model, we randomly selected 200 volumes from the Houston dataset, previously annotated by the Houston graders, and asked the Hadassah graders to determine the presence or absence of cRORA. To assess the level of agreement between the two grading groups, we calculated Fleiss’ *κ* and found a value of 0.65 (CI [0.53, 0.77]), indicating moderate agreement [21]. This finding clearly highlights grader inconsistency as one plausible contributor, alongside other sources of domain shift, to the performance drop mentioned in the previous section, and suggests that inter-grader variability may limit achievable performance gains without changes in annotation consistency or acquisition protocols.

Next, to assess the model’s grader dependency, we calculated the three-way agreement among the two human grader groups and our AI grader. As expected, the level of agreement slightly decreased with the addition of the third grading group, but the reduction was not substantial (*κ*=0.6; CI [0.49, 0.7]), indicating that the model aligns well with both graders. This conclusion is further supported by the pairwise agreement levels between the AI grader and each of the human grader groups (*κ*=0.57; CI [0.42, 0.72] and *κ*=0.56; CI [0.42, 0.68]). Rather than observing a higher *κ* for the AI grader and the human grader group that provided its training data, the similar values indicate comparable level of agreement of the AI grader to both human grader groups and suggest robust generalizability potential for our AI grader.

## Methods

### Datasets

#### The Hadassah dataset

A real-world dataset, composed of volumetric OCT scans (SD-OCT Spectralis, Heidelberg Engineering, Heidelberg, Germany) and associated electronic health records (EHR) data, was obtained from the Hadassah Medical Center’s Department of Ophthalmology. The dataset is part of a longitudinal study on AMD progression over a 13-year period (2009–2022) in an elderly population of consecutive patients. All patients included in this study were over 50 (*μ*=75; *σ*=9) and were diagnosed with AMD. To compile the Hadassah dataset, we randomly selected a subset of eyes, and, for each eye, randomly sampled between one and three OCT volumes from its available scan trajectory, without enforcing fixed temporal spacing. The study adhered to the ethical principles outlined in the declaration of Helsinki. All imaging and EHR data underwent a rigorous de-identification process before being analyzed. Three OCT-certified and masked graders from the Hadassah Reading Center, who underwent extensive training in grading macular diseases with a specific focus on AMD, independently assessed all images according to a standardized reading protocol. Low-quality scans were excluded from further analysis and model development, based on predefined criteria, including severe motion artifacts, truncated macular coverage, or inadequate signal strength preventing reliable assessment of RPE loss or hypertransmission. These exclusions were applied as a quality-control preprocessing step prior to dataset construction, and all dataset sizes reported in this study refer to the final analysis-ready cohorts after this filtering. Because this quality-control filtering was performed as part of routine reading-center preprocessing prior to dataset construction, exact counts of excluded low-quality volumes were not prospectively logged. Unlike the Houston dataset, eyes with additional macular comorbidities (e.g., epiretinal membrane, macular hole, or vascular pathology) were not excluded from the Hadassah cohort, reflecting its longitudinal, real-world design. The graders focused their decisions on findings within the central 3 $m{m}^{2}$ zone centered on the fovea and documented the presence of cRORA at the volume level based on the CAM definitions [2].

The patient subgroups were classified as no AMD, non-nvAMD, and nvAMD [22–25] (see Fig. 1 and the “Grading” section). A systematic validation approach was employed to ensure reliability, consistency, and high-quality standards in the annotated data (see the “Grading” section). The final dataset included 2,504 eyes from 1,531 patients, totaling 3,883 volumes (comprising 131,929 2D B-scans). The annotated OCT volumes in the final dataset varied in the number of 768 × 512 B-scans per volume (*μ*=34; *σ*=11). The scans covered a dimension of 6 × 6 $m{m}^{2}$ on the retina that were centered on the macula, representing an approximate 20-degree field of view. The overall frequency of cRORA in the dataset was 32.6%. Specifically, the recorded frequencies of cRORA in the non-nvAMD and nvAMD subgroups were 24.88% and 50.36%, respectively. Regarding AMD subgroups, the frequencies of no AMD, non-nvAMD, and nvAMD were 12.69%, 46.55%, and 40.76%, respectively.

#### The Houston dataset

The OCT volume data acquired from 1,118 consecutive subjects at the Retina Consultants of Texas Eye Clinics between October 2016 and December 2023 were included in this study and screened for evidence of age-related macular degeneration (AMD). Only one eye per subject was randomly chosen and enrolled in the study. The dataset included Heidelberg Spectralis (HRA+Optical Coherence Tomography OCT SPECTRALIS; Heidelberg Engineering, Inc, Heidelberg, Germany) 6 × 6 $m{m}^{2}$ OCT volumes (fovea centered, 20 × 20 degrees; 49 B-scans spaced 120 microns apart, ART at least of 5). Notably, while both the Hadassah and the Houston datasets were acquired using Heidelberg Spectralis systems, scan density and ART settings were not fully identical between cohorts; the Houston dataset followed a fixed high-density acquisition protocol (49 B-scans, ART ≥ 5), whereas the Hadassah dataset included a broader range of scan densities and spacings reflective of routine clinical practice. The data were transferred to the Doheny Image Reading Research Laboratory (DIRRL) for imaging analysis and annotation. The images were graded by one grader and reviewed by a senior retinal specialist, in compliance with the Declaration of Helsinki. Each volume was assessed for the AMD subgroup (no AMD, non-nvAMD, and nvAMD), and the presence of cRORA. Cases with additional macular diseases or poor-quality imaging were excluded from the analysis. The final Houston dataset comprised 964 analysis-eligible volumes (47,236 2D B-scan slices in total). The recorded cRORA frequency in the whole dataset was 24.37%. The recorded frequencies of cRORA in the non-nvAMD and nvAMD subgroups were 25.08% and 52.63%, respectively. In terms of AMD, the frequencies of the no AMD, non-nvAMD, and nvAMD subgroups were 19.09%, 69.09%, and 11.82%, respectively. Only one eye per subject was included in the Houston dataset to ensure statistical independence between samples, consistent with the original study design and grading workflow.

### Grading

#### Protocol

The cRORA annotation was taken to be any region in which the following were overlapping: (1) the retinal pigment epithelium (RPE) loss > 250 μm; (2) the hypertransmission > 250 μm; and (3) the presence of overlying photoreceptor degeneration [2]. The nvAMD subgroup was classified using both EHR and OCT biomarkers and included both active and inactive nvAMD. Active nvAMD was characterized by choroidal neovascularization (CNV), with features such as subretinal fluid, intraretinal cysts, hyporeflective spaces, subretinal hyperreflective exudate material (SHREM), hyperreflective foci (HRF), and pigment epithelial detachment (PED), along with a need for clinical treatment such as intravitreal anti-VEGF injections [22]. Inactive nvAMD was characterized by previously treated macular neovascularization (MNV) that is stable, with no fluid on OCT for at least six months and no need for further injections [23, 24].

#### Validation

The initial identification of cRORA was performed by the reading center during the primary annotation process. A systematic validation process was employed to ensure the reliability, consistency, and overall quality of the annotated data. To explicitly cross-check annotations between readers, a random 25% sample of the annotated cases was periodically evaluated after completion of each annotation batch through pairwise assessment by two independent graders, using the Intraclass Correlation Coefficient (ICC). In instances where the inter-grader agreement fell below 80%, the final diagnosis was adjudicated by a senior retina specialist (fewer than 5% of batches).

### Model development and analysis

We used the SLIViT model implementation from https://github.com/cozygene/SLIViT. Briefly, this model is a generalization of the vision transformer (ViT) architecture [26]. In contrast to the original ViT, rather than splitting a single image into patches and integrate them through a transformer encoder [27], SLIViT uses a powerful pre-trained feature-map extractor to embed each of the volume slices, and treats their corresponding embeddings as “patches”, for the comprehensive integration in a downstream transformer encoder. As in the original SLIViT paper [13], we initialized the positional embeddings as the number of the original slice and used GeLu as the activation functions [28] in the feature-map integrator. The configurable batch size was set by default to four. For detailed training specifications please refer to section “Model Development and Analysis”.

#### Model hyperparameter tuning

In contrast to the original SLIViT study that conducted a limited hyperparameters tuning experiment, here, we conducted an extensive experiment, evaluating more than 150 model configurations, to enhance our model’s potency. The experiment complied with the following best practices as follows. The original training set was split into sub-train, sub-validation, and sub-test (using the same proportions in the original split). The different examined hyperparameter configurations were evaluated using this split. For each hyperparameter configuration, the weights of the model with the lowest sub-validation loss were used to evaluate the performance on the sub-test set. The hyperparameter configurations examined and their corresponding performance are provided in Supplementary Appendix Table 2.

#### Model training

As described in the SLIViT training protocol, we borrowed an ImageNet-1 K pre-trained ConvNeXt-T backbone, from https://huggingface.co/facebook/convnext-tiny-224, and appended to it a subsequent dense layer to fit a four-category classification task and trained this backbone in a supervised fashion on the publicly available Kermany dataset [19].

During the fine-tuning phase, each 2D B-scan from the OCT volumes was resized to 256 × 256 pixels. Binary cross-entropy was employed as the loss function for the learning task. Since the number of frames per volume varied, we standardized the input by using 19 B-scans per volume. For volumes containing more than 19 frames, equally spaced subsampling was applied. We also experimented with middle subsampling, which selects the central 19 B-scans; however, this approach underperformed compared to equally spaced subsampling (see Supplementary Appendix Table 2). A randomly selected validation set was used to monitor training convergence and determine the optimal model, with the same loss metric applied for test set evaluation. The model weights corresponding to the lowest validation loss were considered the best-performing. To mitigate the risk of overfitting caused by the data split, we implemented five-fold cross-validation and reported the averaged results. The same data splits and validation procedure were used to train and evaluate other benchmark models.

The model was optimized using the default fast.ai optimizer with the default parameters. We considered two fine-tuning approaches that are based on the one-cycle policy [29, 30]. The first was the “end-to-end fine-tuning” (using fast.ai’s fit_one_cycle method), as SLIViT took in the original paper. The second was the “differential fine-tuning” (using fast.ai’s fine_tune method), that is, first unfreezing the final layers of the model and training for a few epochs (one by default) with a low learning rate, and then, unfreezing the rest of the model and end-to-end fine-tuned). For our final model we chose the differential approach that, in our hyperparameter tuning experiment, led to a more performative configuration (see Supplementary Appendix Table 2). While model convergence typically took nearly five epochs, all models were fine-tuned for 10 epochs to further exhaust the model-weights search space. Weights & Biases (https://www.wandb.com*)* was used for experiment tracking and visualizations of the training procedures.

Pre-training the model on the Kermany dataset for 10 epochs and fine-tuning the model on the whole Hadassah dataset for 10 epochs took roughly two hours (each) using a single NVIDIA Tesla V100 Volta GPU Accelerator 32GB Graphics Card. The resulting fine-tuned final checkpoint is readily available at the project’s repository (see Data and Code Availability).

### Benchmarking

We used the 3D version of the ResNet-18 architecture as implemented in https://monai-dev.readthedocs.io/en/stable/networks.html#resnet. We trained the model using fast.ai’s fit_one_cycle method. We used the implementation from SLIViT’s official repository: https://github.com/nrakocz/sliver_net and followed its training instructions. For the 2D ResNet-based model we used the pre-trained checkpoint provided by the authors of the corresponding manuscript. Notably, although this method is evaluated at the volume level, its training occurs at the slice level. Specifically, in the original paper, each training volume with k 2D B-scans had k individual labels (one per slice), and the model was trained accordingly. As a result, we were unable to directly train the model with our volume-level labeled data. Training the model by assigning the volume-level label to each slice did not yield meaningful results (0.74 ROC AUC and 0.5 AP), and thus, we excluded it from the internal evaluation analysis.

### Statistical analysis

The confidence intervals (CIs) included in this study were calculated using a standard bootstrapping procedure with *n* = 1,000 iterations, as employed in other studies [31, 32]. Briefly, let t represent a test set size; for each bootstrap iteration, a set with t samples was randomly drawn-with-replacement from the test set, and a corresponding score was calculated based on the predictions for the sampled set. Given the distribution of the 1,000 bootstrap scores (each corresponds to a single bootstrap iteration), the 50th and 950th ranked scores were selected to determine the 90% CI. To assess the significance level of performance difference between two bootstrap score distributions, we compiled the distribution of score differences (each difference based on scores obtained on the same sampled set). A paired t-test was then conducted on the distribution of score differences (${H}_{0}$: *μ*=0). The improvement was deemed significant if the yielded p-value was lower than 0.01 after a Bonferroni correction application.

## Discussion

In this study we developed a deep-learning model that automates the accurate diagnosis of AMD-related atrophy in 3D OCT scans. We compiled a large labeled dataset of patients with different AMD subgroups defined by neovascular status and leveraged it to train a state-of-the-art architecture for volumetric medical imaging analysis called SLIViT to diagnose cRORA. The trained model is freely available to the community and has the potential to significantly accelerate the rapid, standardized, and scalable annotation of 3D OCT datasets (potentially not only for cRORA but also other retinal biomarkers if used as a fine-tuning starting point), a process that is typically limited by expert time and cost, particularly for longitudinal studies of AMD progression.

Previous studies have investigated the factors contributing to atrophy in patients with AMD [9, 10, 33–35]. However, these studies have focused on detecting cRORA in non-nvAMD, with limited research addressing macular atrophy in nvAMD [3, 4], often yielding inconsistent findings due to the use of different imaging modalities, reliance on clinical trial data, or public databases that do not accurately represent real-world conditions. Additionally, key questions remain unresolved, such as the relationship between atrophy and neovascularization, and how anti-VEGF treatments influence the development of atrophy.

To address these challenges and facilitate broader clinical and research applications, we, in contrast to other previous studies, included scans with a wide spectrum of AMD severity, including patients with advanced neovascular exudation. This way our model is not restricted to a specific AMD subgroup when diagnosing cRORA in OCT images. Our model demonstrates high and consistent performance across diverse clinical scenarios, making it a valuable tool for analyzing the vast number of OCT images collected daily in clinics worldwide.

The model’s outperformance compared to other state-of-the-art models was validated after extensive architecture optimization (through a thorough hyperparameter tuning search). We cross-validated the model on our dataset and demonstrated its superiority over other strong candidate baselines for automating cRORA diagnosis. Following this, we trained the final model and showcased its generalizability and translatability on an external, independent dataset in a zero-shot learning experiment. Notably, the model successfully identified cRORA in patients with nvAMD who had received anti-VEGF injections, underscoring its ability to handle complex cases. This highlights its potential for retrospective studies and research-focused atrophy assessment.

While performance is primarily summarized using ROC AUC and AP metrics, we additionally report sensitivity and specificity for the internal dataset (Appendix Table 1); external evaluation focuses on threshold-independent metrics, reflecting the need for site-specific operating point selection when applying models across heterogeneous real-world datasets. Depending on the intended application, different operating points may be appropriate. For example, in screening-oriented settings, higher sensitivity may be favored to minimize missed cases of cRORA, whereas confirmation or clinical trial enrichment scenarios may prioritize higher specificity to reduce false positives. Accordingly, the relative costs of false negatives and false positives should guide threshold selection in downstream research or clinical workflows. The 19-slice subsampling strategy represents a trade-off between computational efficiency and standardization across datasets, and the potential loss of small or localized lesions, particularly in settings with higher scan density or differing slice spacing across devices and acquisition protocols. Additional sources of domain shift may include ART settings and inclusion criteria such as the presence of other macular pathology.

Although this model shows practical promise, further research is essential to verify its repeatability, effectiveness, and clinical applicability across diverse settings. The training and validation of our model were conducted using OCT scans from the Heidelberg Spectralis, a commonly used device worldwide, which also informed the development of the CAM consensus classification. However, the generalizability of our model to other OCT devices remains uncertain. Furthermore, because the model is confined to regions captured by macular-OCT scans, it does not detect extra-macular atrophic changes that might be identified using other imaging modalities, such as wide-field infrared and autofluorescence imaging. Additionally, the algorithm is limited to classifying cRORA and was not evaluated for the other subtypes of macular atrophies. Nevertheless, a future effort could involve model adjustment given the relevant labels and imaging modalities. Lastly, as with other retrospective volumetric imaging studies, residual within-subject correlation at the volume level cannot be fully eliminated and is acknowledged as a limitation.

Overall, this study marks a significant advancement toward the full automation of AMD-related annotations in OCT scans. By enabling scalable and standardized identification of cRORA across large volumetric datasets, the proposed approach could facilitate retrospective annotation of longitudinal OCT data and serve as a foundation for future studies modeling atrophy development and progression, which would require additional segmentation or regression endpoints and were not evaluated in the present work.

## Supplementary Information

Below is the link to the electronic supplementary material.

Supplementary Material 1

## Data Availability

The fine-tuned model is available at https://tinyurl.com/msu589p4. Publicly available implementations for the models considered in this study were used (please refer to the Model Development and Analysis section for SLIViT and the Benchmarking section for the other models). The 3D OCT B-scan datasets are not publicly accessible due to institutional data use policies and patient privacy concerns, however, they can be obtained from the authors upon reasonable request and with approval from the institutional review board.

## Appendix

**Table 1:** Sensitivity and specificity for cRORA diagnosis (Hadassah dataset).

Subgroup	Sensitivity	Specificity
All (n=587; PP =. 35)	.83 [.81, .86]	.95 [.94, .96]
non-nvAMD (n=235; PP =. 30)	.83 [.79, .87]	.98 [.98, .99]
nvAMD (n=187; PP =. 53)	.83 [.79, .86]	.89 [.87, .92]

Shown are the sensitivity and specificity for cRORA identification evaluated at a fixed reference score threshold of 0.5. The square brackets next to each performance score represent a 90% CI. The test size (n) and the cRORA positive prevalence (PP) for each subgroup are provided in parentheses.
