# Supplementary material for: A deep learning model for automated identification of age-related macular degeneration atrophy
Source: Graefes Arch Clin Exp Ophthalmol. 2026 May 9;264(11):3519–30. doi: 10.1007/s00417-026-07205-4 (PMC13715841; doi:10.1007/s00417-026-07205-4)
Supplement: Supplementary file 1 [file 417_2026_7205_MOESM1_ESM.pdf]

Model configurations and their scores

The configuration with the *lowest loss score* was selected for all downstream analyses in this study. Hyperparameter acronyms were used to prevent table overflow. The hyperparameters keymap is as follows:
fm: Fine-tuning method
ve: ViT Depth
vi: ViT Dim
md: MLP Dim
he: Heads
dr: Dropout
em: Embedding Dropout
sm: Sparsing Method

	**Loss**	ROC AUC	AP	fm	ve	vi	md	he	dr	em	sm
**1**	**0.29876**	0.93602	0.87505	**differential**	**9**	**256**	**512**	**49**	**0.1**	**0**	**eq**
2	0.31316	0.94241	0.86347	end-to-end	9	64	512	19	0.1	0	mid
3	0.31336	0.92409	0.86008	end-to-end	5	128	512	19	0.1	0.1	eq
4	0.31858	0.93748	0.87524	end-to-end	9	64	512	19	0.1	0.1	eq
5	0.32024	0.93308	0.867	end-to-end	9	256	1028	9	0.1	0.1	eq
6	0.32281	0.93478	0.86074	end-to-end	5	64	512	19	0.1	0	mid
7	0.32311	0.93843	0.88469	end-to-end	9	64	256	29	0.1	0	mid
8	0.32348	0.93351	0.86246	end-to-end	9	64	1028	19	0	0	eq
9	0.32495	0.9264	0.87549	differential	5	64	256	39	0	0.1	mid
10	0.33042	0.94255	0.87013	end-to-end	5	64	1028	29	0.1	0.1	mid
11	0.33047	0.93976	0.88201	end-to-end	5	64	1028	19	0	0	eq
12	0.33058	0.93832	0.87326	differential	13	64	1028	9	0.1	0.1	eq
13	0.33164	0.92997	0.86732	end-to-end	1	64	512	29	0.1	0	eq
14	0.33354	0.92334	0.85368	differential	13	128	256	49	0	0	mid
15	0.33728	0.92617	0.86035	differential	13	128	512	39	0.1	0	eq
16	0.33977	0.94045	0.87863	differential	9	64	1028	29	0	0.1	mid
17	0.34255	0.93567	0.86964	end-to-end	9	64	512	9	0	0.1	mid
18	0.34446	0.92853	0.85601	end-to-end	1	128	512	19	0.1	0.1	eq
19	0.3455	0.93449	0.87261	differential	5	128	512	29	0	0	eq
20	0.34746	0.92787	0.87155	end-to-end	1	64	256	19	0	0	eq
21	0.34807	0.93236	0.8747	end-to-end	13	128	256	19	0	0	eq
22	0.34831	0.92962	0.87161	end-to-end	9	128	1028	29	0	0.1	mid
23	0.34851	0.93489	0.86018	differential	5	64	1028	39	0.1	0.1	mid
24	0.3504	0.93293	0.86351	end-to-end	5	64	256	29	0.1	0	mid
25	0.35062	0.93089	0.85593	differential	9	64	1028	29	0	0	eq
26	0.35162	0.91569	0.84355	end-to-end	5	64	512	19	0	0	mid
27	0.35399	0.92715	0.85474	end-to-end	9	64	512	19	0	0	mid
28	0.35448	0.92643	0.85412	end-to-end	9	64	512	19	0	0.1	eq
29	0.35469	0.93348	0.85564	differential	5	256	256	49	0	0	mid
30	0.3561	0.9302	0.86412	differential	5	128	256	29	0	0	mid
31	0.35652	0.91615	0.82906	differential	5	256	512	49	0	0	eq
32	0.3575	0.93884	0.86703	end-to-end	5	64	1028	49	0	0	mid
33	0.35852	0.9321	0.85365	end-to-end	9	256	512	29	0.1	0.1	mid

Continued on next page

34	0.36032	0.93702	0.87341	differential	13	128	512	29	0.1	0.1	eq
35	0.36035	0.93936	0.88078	end-to-end	5	128	512	49	0.1	0	mid
36	0.36038	0.93066	0.86983	end-to-end	1	64	1028	19	0	0	eq
37	0.3615	0.92372	0.85147	end-to-end	1	64	1028	9	0.1	0	eq
38	0.36164	0.94045	0.88533	end-to-end	9	64	512	19	0.1	0	eq
39	0.3632	0.92277	0.809	differential	9	64	512	9	0	0.1	eq
40	0.36429	0.93147	0.85828	end-to-end	1	64	512	19	0	0	mid
41	0.36535	0.91583	0.85943	differential	13	128	256	19	0	0	mid
42	0.36705	0.94367	0.89035	end-to-end	1	256	256	19	0	0	eq
43	0.36757	0.9365	0.87183	differential	5	256	512	49	0.1	0	mid
44	0.36761	0.92945	0.85996	end-to-end	1	64	512	9	0	0.1	mid
45	0.36774	0.93155	0.87162	end-to-end	1	128	1028	29	0	0.1	eq
46	0.36787	0.92582	0.85242	end-to-end	1	64	256	29	0.1	0	mid
47	0.36869	0.93296	0.86747	end-to-end	5	128	1028	29	0	0.1	eq
48	0.36915	0.92859	0.85658	end-to-end	9	128	512	9	0	0.1	eq
49	0.37107	0.90967	0.80035	end-to-end	1	64	256	9	0	0.1	eq
50	0.3713	0.92962	0.85838	differential	9	128	1028	9	0	0	eq
51	0.37145	0.91053	0.8047	differential	13	128	512	9	0	0.1	mid
52	0.3729	0.92084	0.85041	end-to-end	9	256	1028	9	0	0.1	eq
53	0.3736	0.92867	0.82491	end-to-end	5	64	512	9	0	0.1	mid
54	0.37423	0.91822	0.83502	end-to-end	9	64	1028	19	0	0.1	eq
55	0.37556	0.92836	0.85133	differential	5	256	512	49	0	0.1	eq
56	0.37597	0.93299	0.87284	differential	5	256	1028	19	0.1	0.1	eq
57	0.37742	0.92553	0.84566	end-to-end	9	128	512	19	0.1	0.1	eq
58	0.37805	0.92421	0.85567	end-to-end	1	64	512	29	0	0	eq
59	0.37811	0.93861	0.87309	differential	9	128	256	29	0.1	0.1	eq
60	0.37815	0.92784	0.85917	end-to-end	5	64	256	9	0	0.1	eq
61	0.37866	0.91332	0.85447	differential	9	128	256	19	0.1	0	eq
62	0.37871	0.93144	0.86234	differential	13	64	512	29	0	0.1	mid
63	0.37887	0.92067	0.7836	differential	9	64	512	49	0.1	0.1	mid
64	0.38004	0.93613	0.85311	end-to-end	1	64	512	19	0.1	0	mid
65	0.38139	0.93098	0.85692	differential	9	128	1028	39	0	0.1	eq
66	0.38229	0.9124	0.85214	end-to-end	5	128	512	9	0.1	0.1	eq
67	0.38285	0.90414	0.79962	end-to-end	5	256	512	9	0.1	0	mid
68	0.38444	0.90817	0.80686	end-to-end	5	64	256	9	0	0	mid
69	0.38457	0.93103	0.86614	differential	9	128	1028	39	0.1	0	eq
70	0.38488	0.92363	0.86391	end-to-end	5	256	512	29	0	0	eq
71	0.38951	0.93363	0.86768	end-to-end	1	256	512	29	0.1	0	eq
72	0.38963	0.92435	0.8527	end-to-end	1	64	512	19	0	0.1	eq
73	0.39065	0.92081	0.838	end-to-end	5	64	512	29	0	0	eq
74	0.39183	0.91713	0.86506	differential	5	128	512	39	0.1	0	mid
75	0.39269	0.91615	0.84743	end-to-end	9	64	1028	19	0.1	0.1	mid
76	0.3939	0.93642	0.87958	differential	13	256	256	49	0	0	eq
77	0.39518	0.93688	0.87809	end-to-end	1	128	256	9	0.1	0.1	eq
78	0.39636	0.92887	0.8475	end-to-end	1	64	1028	29	0	0.1	mid
79	0.39667	0.92507	0.83268	end-to-end	5	64	512	19	0	0.1	eq
80	0.39705	0.92297	0.85309	end-to-end	5	128	256	9	0	0.1	mid

Continued on next page

81	0.3971	0.9181	0.84112	end-to-end	9	256	512	9	0	0.1	mid
82	0.39719	0.93351	0.86319	differential	13	128	256	29	0.1	0.1	eq
83	0.39736	0.92908	0.87511	end-to-end	9	64	512	29	0	0	eq
84	0.39913	0.89691	0.798	differential	5	256	512	29	0.1	0.1	eq
85	0.40031	0.9217	0.8416	end-to-end	9	256	512	29	0	0.1	mid
86	0.40248	0.93363	0.86013	end-to-end	9	64	1028	29	0.1	0.1	mid
87	0.40298	0.91969	0.85767	end-to-end	9	256	256	19	0	0	eq
88	0.4037	0.93391	0.86639	end-to-end	5	128	256	9	0.1	0.1	eq
89	0.40464	0.9232	0.83915	differential	5	256	512	39	0	0.1	eq
90	0.40513	0.91289	0.80979	differential	9	128	1028	49	0.1	0.1	eq
91	0.4062	0.92412	0.85699	end-to-end	9	256	512	29	0.1	0	eq
92	0.40635	0.93368	0.83309	differential	13	256	1028	39	0	0	mid
93	0.40697	0.92951	0.85907	differential	13	128	256	19	0	0	eq
94	0.40813	0.9145	0.84541	end-to-end	13	256	512	49	0	0	eq
95	0.40847	0.92867	0.84954	end-to-end	5	256	1028	9	0.1	0.1	eq
96	0.40936	0.93299	0.86903	end-to-end	5	64	512	29	0.1	0	eq
97	0.4094	0.9433	0.88814	end-to-end	5	64	1028	9	0.1	0	eq
98	0.41321	0.92873	0.86947	end-to-end	9	256	512	9	0	0.1	eq
99	0.41322	0.88945	0.76381	end-to-end	5	128	512	9	0	0.1	eq
100	0.4134	0.92061	0.83212	end-to-end	9	256	512	19	0	0	mid
101	0.41571	0.92257	0.83139	end-to-end	1	256	512	9	0.1	0	mid
102	0.41639	0.91894	0.83692	end-to-end	1	128	256	9	0	0.1	mid
103	0.41659	0.93898	0.87511	end-to-end	9	64	256	19	0	0	eq
104	0.41798	0.91404	0.85006	end-to-end	9	256	1028	29	0	0.1	mid
105	0.42147	0.91214	0.78352	end-to-end	1	128	512	9	0.1	0.1	eq
106	0.42149	0.93541	0.85282	differential	9	256	1028	19	0.1	0	eq
107	0.42317	0.92139	0.82295	end-to-end	9	64	1028	9	0.1	0	eq
108	0.42444	0.91275	0.82388	end-to-end	1	64	1028	29	0.1	0.1	mid
109	0.42656	0.92415	0.85915	end-to-end	9	128	512	9	0.1	0.1	eq
110	0.42707	0.93175	0.86632	differential	5	256	256	29	0.1	0	eq
111	0.42782	0.91456	0.81088	end-to-end	9	128	256	19	0.1	0.1	mid
112	0.4279	0.92228	0.85378	end-to-end	9	128	256	9	0.1	0	eq
113	0.43069	0.89567	0.8095	end-to-end	9	128	512	19	0	0.1	eq
114	0.43069	0.92723	0.86838	end-to-end	5	256	512	9	0	0.1	eq
115	0.43074	0.92395	0.85603	end-to-end	5	256	1028	19	0	0.1	mid
116	0.43075	0.92519	0.83036	end-to-end	9	64	256	9	0	0	mid
117	0.43511	0.88957	0.7835	end-to-end	1	256	1028	19	0	0.1	mid
118	0.43651	0.9103	0.81062	end-to-end	1	256	1028	19	0	0	mid
119	0.44245	0.89564	0.84354	differential	13	128	256	19	0	0.1	mid
120	0.44299	0.89417	0.7764	end-to-end	5	256	1028	19	0	0	mid
121	0.44423	0.93129	0.86264	end-to-end	9	256	1028	19	0	0	mid
122	0.45513	0.89743	0.79089	end-to-end	9	128	1028	9	0	0	eq
123	0.45944	0.87922	0.78103	end-to-end	9	256	1028	19	0	0.1	mid
124	0.46144	0.84727	0.69877	end-to-end	1	256	512	19	0	0	mid
125	0.46325	0.9374	0.87653	end-to-end	9	64	1028	29	0	0.1	mid
126	0.46653	0.92925	0.85634	end-to-end	5	256	512	29	0.1	0	eq
127	0.46794	0.91903	0.81523	end-to-end	1	256	1028	29	0	0.1	mid

Continued on next page

128	0.47045	0.83978	0.71128	differential	13	64	512	9	0	0	mid
129	0.47074	0.92614	0.86083	end-to-end	9	256	512	29	0	0	eq
130	0.4731	0.89714	0.73692	differential	5	64	512	49	0	0	eq
131	0.47723	0.91491	0.8346	end-to-end	9	128	1028	29	0	0.1	eq
132	0.47728	0.92913	0.86319	end-to-end	5	256	256	19	0	0	eq
133	0.47832	0.92162	0.84436	end-to-end	9	128	1028	29	0.1	0.1	eq
134	0.48659	0.91992	0.80498	end-to-end	1	256	512	9	0	0.1	eq
135	0.48793	0.91816	0.8535	end-to-end	9	256	512	9	0.1	0	mid
136	0.4969	0.92188	0.83503	end-to-end	9	128	256	19	0.1	0.1	eq
137	0.52444	0.77951	0.53287	end-to-end	5	256	1028	29	0	0.1	mid
138	0.53076	0.9281	0.86464	end-to-end	9	256	512	9	0	0	eq
139	0.54179	0.91641	0.84142	end-to-end	1	128	512	9	0	0.1	eq
140	0.58949	0.76684	0.53307	end-to-end	9	128	256	9	0	0.1	mid
141	0.60465	0.62771	0.37925	end-to-end	1	256	1028	9	0	0.1	eq
142	0.60734	0.62772	0.39728	differential	5	64	1028	39	0	0	eq
143	0.60744	0.59251	0.34842	end-to-end	9	64	256	9	0	0.1	eq
144	0.60758	0.71168	0.43569	end-to-end	5	64	256	19	0	0	eq
145	0.60791	0.61645	0.36771	differential	13	256	512	9	0	0.1	eq
146	0.60793	0.58388	0.33507	end-to-end	5	256	512	19	0	0	mid
147	0.60796	0.4525	0.26321	end-to-end	9	128	256	9	0.1	0.1	eq
148	0.60804	0.63119	0.42174	end-to-end	5	64	1028	29	0	0.1	mid
149	0.60806	0.53978	0.33374	differential	5	256	1028	9	0	0.1	eq
150	0.60806	0.69388	0.42031	differential	13	128	512	39	0	0.1	eq
151	0.60818	0.56014	0.32715	end-to-end	1	64	256	9	0	0	mid
152	0.60822	0.71829	0.43185	end-to-end	9	64	512	29	0.1	0	eq
153	0.60836	0.59618	0.36764	end-to-end	1	256	1028	9	0.1	0.1	eq
154	0.60872	0.68428	0.41479	differential	13	128	256	29	0	0	mid
155	0.60916	0.56525	0.31619	end-to-end	5	256	1028	9	0	0.1	eq
156	0.60929	0.59259	0.34192	end-to-end	9	128	512	29	0.1	0.1	eq
157	0.60929	0.59259	0.34192	end-to-end	9	128	512	29	0.1	0.1	eq
158	0.60916	0.56525	0.31619	end-to-end	5	256	1028	9	0	0.1	eq
