# Supplementary material for: A deep learning model for automated identification of age-related macular degeneration atrophy
Source: Graefes Arch Clin Exp Ophthalmol. Author manuscript; Available in PMC 2026 Oct 28. (PMC13715841; doi:10.1007/s00417-026-07205-4)
Supplement: Supplementary file 1 [file NIHMS2214838-supplement-Supplementary_material_1.pdf]

**Model configurations and their scores**

**The configuration with the *lowest loss score*** was selected for all downstream analyses in this study. Hyperparameter acronyms were used to prevent table overflow. The hyperparameters keymap is as follows:
fm: Fine-tuning method
ve: ViT Depth
vi: ViT Dim
md: MLP Dim
he: Heads
dr: Dropout
em: Embedding Dropout
sm: Sparsing Method

| | **Loss** | ROC AUC | AP | fm | ve | vi | md | he | dr | em | sm |
|---|---|---|---|---|---|---|---|---|---|---|---|
| **1** | **0.29876** | 0.93602 | 0.87505 | **differential** | **9** | **256** | **512** | **49** | **0.1** | **0** | **eq** |
| 2 | 0.31316 | 0.94241 | 0.86347 | end-to-end | 9 | 64 | 512 | 19 | 0.1 | 0 | mid |
| 3 | 0.31336 | 0.92409 | 0.86008 | end-to-end | 5 | 128 | 512 | 19 | 0.1 | 0.1 | eq |
| 4 | 0.31858 | 0.93748 | 0.87524 | end-to-end | 9 | 64 | 512 | 19 | 0.1 | 0.1 | eq |
| 5 | 0.32024 | 0.93308 | 0.867 | end-to-end | 9 | 256 | 1028 | 9 | 0.1 | 0.1 | eq |
| 6 | 0.32281 | 0.93478 | 0.86074 | end-to-end | 5 | 64 | 512 | 19 | 0.1 | 0 | mid |
| 7 | 0.32311 | 0.93843 | 0.88469 | end-to-end | 9 | 64 | 256 | 29 | 0.1 | 0 | mid |
| 8 | 0.32348 | 0.93351 | 0.86246 | end-to-end | 9 | 64 | 1028 | 19 | 0 | 0 | eq |
| 9 | 0.32495 | 0.9264 | 0.87549 | differential | 5 | 64 | 256 | 39 | 0 | 0.1 | mid |
| 10 | 0.33042 | 0.94255 | 0.87013 | end-to-end | 5 | 64 | 1028 | 29 | 0.1 | 0.1 | mid |
| 11 | 0.33047 | 0.93976 | 0.88201 | end-to-end | 5 | 64 | 1028 | 19 | 0 | 0 | eq |
| 12 | 0.33058 | 0.93832 | 0.87326 | differential | 13 | 64 | 1028 | 9 | 0.1 | 0.1 | eq |
| 13 | 0.33164 | 0.92997 | 0.86732 | end-to-end | 1 | 64 | 512 | 29 | 0.1 | 0 | eq |
| 14 | 0.33354 | 0.92334 | 0.85368 | differential | 13 | 128 | 256 | 49 | 0 | 0 | mid |
| 15 | 0.33728 | 0.92617 | 0.86035 | differential | 13 | 128 | 512 | 39 | 0.1 | 0 | eq |
| 16 | 0.33977 | 0.94045 | 0.87863 | differential | 9 | 64 | 1028 | 29 | 0 | 0.1 | mid |
| 17 | 0.34255 | 0.93567 | 0.86964 | end-to-end | 9 | 64 | 512 | 9 | 0 | 0.1 | mid |
| 18 | 0.34446 | 0.92853 | 0.85601 | end-to-end | 1 | 128 | 512 | 19 | 0.1 | 0.1 | eq |
| 19 | 0.3455 | 0.93449 | 0.87261 | differential | 5 | 128 | 512 | 29 | 0 | 0 | eq |
| 20 | 0.34746 | 0.92787 | 0.87155 | end-to-end | 1 | 64 | 256 | 19 | 0 | 0 | eq |
| 21 | 0.34807 | 0.93236 | 0.8747 | end-to-end | 13 | 128 | 256 | 19 | 0 | 0 | eq |
| 22 | 0.34831 | 0.92962 | 0.87161 | end-to-end | 9 | 128 | 1028 | 29 | 0 | 0.1 | mid |
| 23 | 0.34851 | 0.93489 | 0.86018 | differential | 5 | 64 | 1028 | 39 | 0.1 | 0.1 | mid |
| 24 | 0.3504 | 0.93293 | 0.86351 | end-to-end | 5 | 64 | 256 | 29 | 0.1 | 0 | mid |
| 25 | 0.35062 | 0.93089 | 0.85593 | differential | 9 | 64 | 1028 | 29 | 0 | 0 | eq |
| 26 | 0.35162 | 0.91569 | 0.84355 | end-to-end | 5 | 64 | 512 | 19 | 0 | 0 | mid |
| 27 | 0.35399 | 0.92715 | 0.85474 | end-to-end | 9 | 64 | 512 | 19 | 0 | 0 | mid |
| 28 | 0.35448 | 0.92643 | 0.85412 | end-to-end | 9 | 64 | 512 | 19 | 0 | 0.1 | eq |
| 29 | 0.35469 | 0.93348 | 0.85564 | differential | 5 | 256 | 256 | 49 | 0 | 0 | mid |
| 30 | 0.3561 | 0.9302 | 0.86412 | differential | 5 | 128 | 256 | 29 | 0 | 0 | mid |
| 31 | 0.35652 | 0.91615 | 0.82906 | differential | 5 | 256 | 512 | 49 | 0 | 0 | eq |
| 32 | 0.3575 | 0.93884 | 0.86703 | end-to-end | 5 | 64 | 1028 | 49 | 0 | 0 | mid |
| 33 | 0.35852 | 0.9321 | 0.85365 | end-to-end | 9 | 256 | 512 | 29 | 0.1 | 0.1 | mid |

Continued on next page

| | | | | | | | | | | | |
|---|---|---|---|---|---|---|---|---|---|---|---|
| 34 | 0.36032 | 0.93702 | 0.87341 | differential | 13 | 128 | 512 | 29 | 0.1 | 0.1 | eq |
| 35 | 0.36035 | 0.93936 | 0.88078 | end-to-end | 5 | 128 | 512 | 49 | 0.1 | 0 | mid |
| 36 | 0.36038 | 0.93066 | 0.86983 | end-to-end | 1 | 64 | 1028 | 19 | 0 | 0 | eq |
| 37 | 0.3615 | 0.92372 | 0.85147 | end-to-end | 1 | 64 | 1028 | 9 | 0.1 | 0 | eq |
| 38 | 0.36164 | 0.94045 | 0.88533 | end-to-end | 9 | 64 | 512 | 19 | 0.1 | 0 | eq |
| 39 | 0.3632 | 0.92277 | 0.809 | differential | 9 | 64 | 512 | 9 | 0 | 0.1 | eq |
| 40 | 0.36429 | 0.93147 | 0.85828 | end-to-end | 1 | 64 | 512 | 19 | 0 | 0 | mid |
| 41 | 0.36535 | 0.91583 | 0.85943 | differential | 13 | 128 | 256 | 19 | 0 | 0 | mid |
| 42 | 0.36705 | 0.94367 | 0.89035 | end-to-end | 1 | 256 | 256 | 19 | 0 | 0 | eq |
| 43 | 0.36757 | 0.9365 | 0.87183 | differential | 5 | 256 | 512 | 49 | 0.1 | 0 | mid |
| 44 | 0.36761 | 0.92945 | 0.85996 | end-to-end | 1 | 64 | 512 | 9 | 0 | 0.1 | mid |
| 45 | 0.36774 | 0.93155 | 0.87162 | end-to-end | 1 | 128 | 1028 | 29 | 0 | 0.1 | eq |
| 46 | 0.36787 | 0.92582 | 0.85242 | end-to-end | 1 | 64 | 256 | 29 | 0.1 | 0 | mid |
| 47 | 0.36869 | 0.93296 | 0.86747 | end-to-end | 5 | 128 | 1028 | 29 | 0 | 0.1 | eq |
| 48 | 0.36915 | 0.92859 | 0.85658 | end-to-end | 9 | 128 | 512 | 9 | 0 | 0.1 | eq |
| 49 | 0.37107 | 0.90967 | 0.80035 | end-to-end | 1 | 64 | 256 | 9 | 0 | 0.1 | eq |
| 50 | 0.3713 | 0.92962 | 0.85838 | differential | 9 | 128 | 1028 | 9 | 0 | 0 | eq |
| 51 | 0.37145 | 0.91053 | 0.8047 | differential | 13 | 128 | 512 | 9 | 0 | 0.1 | mid |
| 52 | 0.3729 | 0.92084 | 0.85041 | end-to-end | 9 | 256 | 1028 | 9 | 0 | 0.1 | eq |
| 53 | 0.3736 | 0.92867 | 0.82491 | end-to-end | 5 | 64 | 512 | 9 | 0 | 0.1 | mid |
| 54 | 0.37423 | 0.91822 | 0.83502 | end-to-end | 9 | 64 | 1028 | 19 | 0 | 0.1 | eq |
| 55 | 0.37556 | 0.92836 | 0.85133 | differential | 5 | 256 | 512 | 49 | 0 | 0.1 | eq |
| 56 | 0.37597 | 0.93299 | 0.87284 | differential | 5 | 256 | 1028 | 19 | 0.1 | 0.1 | eq |
| 57 | 0.37742 | 0.92553 | 0.84566 | end-to-end | 9 | 128 | 512 | 19 | 0.1 | 0.1 | eq |
| 58 | 0.37805 | 0.92421 | 0.85567 | end-to-end | 1 | 64 | 512 | 29 | 0 | 0 | eq |
| 59 | 0.37811 | 0.93861 | 0.87309 | differential | 9 | 128 | 256 | 29 | 0.1 | 0.1 | eq |
| 60 | 0.37815 | 0.92784 | 0.85917 | end-to-end | 5 | 64 | 256 | 9 | 0 | 0.1 | eq |
| 61 | 0.37866 | 0.91332 | 0.85447 | differential | 9 | 128 | 256 | 19 | 0.1 | 0 | eq |
| 62 | 0.37871 | 0.93144 | 0.86234 | differential | 13 | 64 | 512 | 29 | 0 | 0.1 | mid |
| 63 | 0.37887 | 0.92067 | 0.7836 | differential | 9 | 64 | 512 | 49 | 0.1 | 0.1 | mid |
| 64 | 0.38004 | 0.93613 | 0.85311 | end-to-end | 1 | 64 | 512 | 19 | 0.1 | 0 | mid |
| 65 | 0.38139 | 0.93098 | 0.85692 | differential | 9 | 128 | 1028 | 39 | 0 | 0.1 | eq |
| 66 | 0.38229 | 0.9124 | 0.85214 | end-to-end | 5 | 128 | 512 | 9 | 0.1 | 0.1 | eq |
| 67 | 0.38285 | 0.90414 | 0.79962 | end-to-end | 5 | 256 | 512 | 9 | 0.1 | 0 | mid |
| 68 | 0.38444 | 0.90817 | 0.80686 | end-to-end | 5 | 64 | 256 | 9 | 0 | 0 | mid |
| 69 | 0.38457 | 0.93103 | 0.86614 | differential | 9 | 128 | 1028 | 39 | 0.1 | 0 | eq |
| 70 | 0.38488 | 0.92363 | 0.86391 | end-to-end | 5 | 256 | 512 | 29 | 0 | 0 | eq |
| 71 | 0.38951 | 0.93363 | 0.86768 | end-to-end | 1 | 256 | 512 | 29 | 0.1 | 0 | eq |
| 72 | 0.38963 | 0.92435 | 0.8527 | end-to-end | 1 | 64 | 512 | 19 | 0 | 0.1 | eq |
| 73 | 0.39065 | 0.92081 | 0.838 | end-to-end | 5 | 64 | 512 | 29 | 0 | 0 | eq |
| 74 | 0.39183 | 0.91713 | 0.86506 | differential | 5 | 128 | 512 | 39 | 0.1 | 0 | mid |
| 75 | 0.39269 | 0.91615 | 0.84743 | end-to-end | 9 | 64 | 1028 | 19 | 0.1 | 0.1 | mid |
| 76 | 0.3939 | 0.93642 | 0.87958 | differential | 13 | 256 | 256 | 49 | 0 | 0 | eq |
| 77 | 0.39518 | 0.93688 | 0.87809 | end-to-end | 1 | 128 | 256 | 9 | 0.1 | 0.1 | eq |
| 78 | 0.39636 | 0.92887 | 0.8475 | end-to-end | 1 | 64 | 1028 | 29 | 0 | 0.1 | mid |
| 79 | 0.39667 | 0.92507 | 0.83268 | end-to-end | 5 | 64 | 512 | 19 | 0 | 0.1 | eq |
| 80 | 0.39705 | 0.92297 | 0.85309 | end-to-end | 5 | 128 | 256 | 9 | 0 | 0.1 | mid |

Continued on next page

| | | | | | | | | | | | |
|---|---|---|---|---|---|---|---|---|---|---|---|
| 81 | 0.3971 | 0.9181 | 0.84112 | end-to-end | 9 | 256 | 512 | 9 | 0 | 0.1 | mid |
| 82 | 0.39719 | 0.93351 | 0.86319 | differential | 13 | 128 | 256 | 29 | 0.1 | 0.1 | eq |
| 83 | 0.39736 | 0.92908 | 0.87511 | end-to-end | 9 | 64 | 512 | 29 | 0 | 0 | eq |
| 84 | 0.39913 | 0.89691 | 0.798 | differential | 5 | 256 | 512 | 29 | 0.1 | 0.1 | eq |
| 85 | 0.40031 | 0.9217 | 0.8416 | end-to-end | 9 | 256 | 512 | 29 | 0 | 0.1 | mid |
| 86 | 0.40248 | 0.93363 | 0.86013 | end-to-end | 9 | 64 | 1028 | 29 | 0.1 | 0.1 | mid |
| 87 | 0.40298 | 0.91969 | 0.85767 | end-to-end | 9 | 256 | 256 | 19 | 0 | 0 | eq |
| 88 | 0.4037 | 0.93391 | 0.86639 | end-to-end | 5 | 128 | 256 | 9 | 0.1 | 0.1 | eq |
| 89 | 0.40464 | 0.9232 | 0.83915 | differential | 5 | 256 | 512 | 39 | 0 | 0.1 | eq |
| 90 | 0.40513 | 0.91289 | 0.80979 | differential | 9 | 128 | 1028 | 49 | 0.1 | 0.1 | eq |
| 91 | 0.4062 | 0.92412 | 0.85699 | end-to-end | 9 | 256 | 512 | 29 | 0.1 | 0 | eq |
| 92 | 0.40635 | 0.93368 | 0.83309 | differential | 13 | 256 | 1028 | 39 | 0 | 0 | mid |
| 93 | 0.40697 | 0.92951 | 0.85907 | differential | 13 | 128 | 256 | 19 | 0 | 0 | eq |
| 94 | 0.40813 | 0.9145 | 0.84541 | end-to-end | 13 | 256 | 512 | 49 | 0 | 0 | eq |
| 95 | 0.40847 | 0.92867 | 0.84954 | end-to-end | 5 | 256 | 1028 | 9 | 0.1 | 0.1 | eq |
| 96 | 0.40936 | 0.93299 | 0.86903 | end-to-end | 5 | 64 | 512 | 29 | 0.1 | 0 | eq |
| 97 | 0.4094 | 0.9433 | 0.88814 | end-to-end | 5 | 64 | 1028 | 9 | 0.1 | 0 | eq |
| 98 | 0.41321 | 0.92873 | 0.86947 | end-to-end | 9 | 256 | 512 | 9 | 0 | 0.1 | eq |
| 99 | 0.41322 | 0.88945 | 0.76381 | end-to-end | 5 | 128 | 512 | 9 | 0 | 0.1 | eq |
| 100 | 0.4134 | 0.92061 | 0.83212 | end-to-end | 9 | 256 | 512 | 19 | 0 | 0 | mid |
| 101 | 0.41571 | 0.92257 | 0.83139 | end-to-end | 1 | 256 | 512 | 9 | 0.1 | 0 | mid |
| 102 | 0.41639 | 0.91894 | 0.83692 | end-to-end | 1 | 128 | 256 | 9 | 0 | 0.1 | mid |
| 103 | 0.41659 | 0.93898 | 0.87511 | end-to-end | 9 | 64 | 256 | 19 | 0 | 0 | eq |
| 104 | 0.41798 | 0.91404 | 0.85006 | end-to-end | 9 | 256 | 1028 | 29 | 0 | 0.1 | mid |
| 105 | 0.42147 | 0.91214 | 0.78352 | end-to-end | 1 | 128 | 512 | 9 | 0.1 | 0.1 | eq |
| 106 | 0.42149 | 0.93541 | 0.85282 | differential | 9 | 256 | 1028 | 19 | 0.1 | 0 | eq |
| 107 | 0.42317 | 0.92139 | 0.82295 | end-to-end | 9 | 64 | 1028 | 9 | 0.1 | 0 | eq |
| 108 | 0.42444 | 0.91275 | 0.82388 | end-to-end | 1 | 64 | 1028 | 29 | 0.1 | 0.1 | mid |
| 109 | 0.42656 | 0.92415 | 0.85915 | end-to-end | 9 | 128 | 512 | 9 | 0.1 | 0.1 | eq |
| 110 | 0.42707 | 0.93175 | 0.86632 | differential | 5 | 256 | 256 | 29 | 0.1 | 0 | eq |
| 111 | 0.42782 | 0.91456 | 0.81088 | end-to-end | 9 | 128 | 256 | 19 | 0.1 | 0.1 | mid |
| 112 | 0.4279 | 0.92228 | 0.85378 | end-to-end | 9 | 128 | 256 | 9 | 0.1 | 0 | eq |
| 113 | 0.43069 | 0.89567 | 0.8095 | end-to-end | 9 | 128 | 512 | 19 | 0 | 0.1 | eq |
| 114 | 0.43069 | 0.92723 | 0.86838 | end-to-end | 5 | 256 | 512 | 9 | 0 | 0.1 | eq |
| 115 | 0.43074 | 0.92395 | 0.85603 | end-to-end | 5 | 256 | 1028 | 19 | 0 | 0.1 | mid |
| 116 | 0.43075 | 0.92519 | 0.83036 | end-to-end | 9 | 64 | 256 | 9 | 0 | 0 | mid |
| 117 | 0.43511 | 0.88957 | 0.7835 | end-to-end | 1 | 256 | 1028 | 19 | 0 | 0.1 | mid |
| 118 | 0.43651 | 0.9103 | 0.81062 | end-to-end | 1 | 256 | 1028 | 19 | 0 | 0 | mid |
| 119 | 0.44245 | 0.89564 | 0.84354 | differential | 13 | 128 | 256 | 19 | 0 | 0.1 | mid |
| 120 | 0.44299 | 0.89417 | 0.7764 | end-to-end | 5 | 256 | 1028 | 19 | 0 | 0 | mid |
| 121 | 0.44423 | 0.93129 | 0.86264 | end-to-end | 9 | 256 | 1028 | 19 | 0 | 0 | mid |
| 122 | 0.45513 | 0.89743 | 0.79089 | end-to-end | 9 | 128 | 1028 | 9 | 0 | 0 | eq |
| 123 | 0.45944 | 0.87922 | 0.78103 | end-to-end | 9 | 256 | 1028 | 19 | 0 | 0.1 | mid |
| 124 | 0.46144 | 0.84727 | 0.69877 | end-to-end | 1 | 256 | 512 | 19 | 0 | 0 | mid |
| 125 | 0.46325 | 0.9374 | 0.87653 | end-to-end | 9 | 64 | 1028 | 29 | 0 | 0.1 | mid |
| 126 | 0.46653 | 0.92925 | 0.85634 | end-to-end | 5 | 256 | 512 | 29 | 0.1 | 0 | eq |
| 127 | 0.46794 | 0.91903 | 0.81523 | end-to-end | 1 | 256 | 1028 | 29 | 0 | 0.1 | mid |

Continued on next page

| | | | | | | | | | | | |
|---|---|---|---|---|---|---|---|---|---|---|---|
| 128 | 0.47045 | 0.83978 | 0.71128 | differential | 13 | 64 | 512 | 9 | 0 | 0 | mid |
| 129 | 0.47074 | 0.92614 | 0.86083 | end-to-end | 9 | 256 | 512 | 29 | 0 | 0 | eq |
| 130 | 0.4731 | 0.89714 | 0.73692 | differential | 5 | 64 | 512 | 49 | 0 | 0 | eq |
| 131 | 0.47723 | 0.91491 | 0.8346 | end-to-end | 9 | 128 | 1028 | 29 | 0 | 0.1 | eq |
| 132 | 0.47728 | 0.92913 | 0.86319 | end-to-end | 5 | 256 | 256 | 19 | 0 | 0 | eq |
| 133 | 0.47832 | 0.92162 | 0.84436 | end-to-end | 9 | 128 | 1028 | 29 | 0.1 | 0.1 | eq |
| 134 | 0.48659 | 0.91992 | 0.80498 | end-to-end | 1 | 256 | 512 | 9 | 0 | 0.1 | eq |
| 135 | 0.48793 | 0.91816 | 0.8535 | end-to-end | 9 | 256 | 512 | 9 | 0.1 | 0 | mid |
| 136 | 0.4969 | 0.92188 | 0.83503 | end-to-end | 9 | 128 | 256 | 19 | 0.1 | 0.1 | eq |
| 137 | 0.52444 | 0.77951 | 0.53287 | end-to-end | 5 | 256 | 1028 | 29 | 0 | 0.1 | mid |
| 138 | 0.53076 | 0.9281 | 0.86464 | end-to-end | 9 | 256 | 512 | 9 | 0 | 0 | eq |
| 139 | 0.54179 | 0.91641 | 0.84142 | end-to-end | 1 | 128 | 512 | 9 | 0 | 0.1 | eq |
| 140 | 0.58949 | 0.76684 | 0.53307 | end-to-end | 9 | 128 | 256 | 9 | 0 | 0.1 | mid |
| 141 | 0.60465 | 0.62771 | 0.37925 | end-to-end | 1 | 256 | 1028 | 9 | 0 | 0.1 | eq |
| 142 | 0.60734 | 0.62772 | 0.39728 | differential | 5 | 64 | 1028 | 39 | 0 | 0 | eq |
| 143 | 0.60744 | 0.59251 | 0.34842 | end-to-end | 9 | 64 | 256 | 9 | 0 | 0.1 | eq |
| 144 | 0.60758 | 0.71168 | 0.43569 | end-to-end | 5 | 64 | 256 | 19 | 0 | 0 | eq |
| 145 | 0.60791 | 0.61645 | 0.36771 | differential | 13 | 256 | 512 | 9 | 0 | 0.1 | eq |
| 146 | 0.60793 | 0.58388 | 0.33507 | end-to-end | 5 | 256 | 512 | 19 | 0 | 0 | mid |
| 147 | 0.60796 | 0.4525 | 0.26321 | end-to-end | 9 | 128 | 256 | 9 | 0.1 | 0.1 | eq |
| 148 | 0.60804 | 0.63119 | 0.42174 | end-to-end | 5 | 64 | 1028 | 29 | 0 | 0.1 | mid |
| 149 | 0.60806 | 0.53978 | 0.33374 | differential | 5 | 256 | 1028 | 9 | 0 | 0.1 | eq |
| 150 | 0.60806 | 0.69388 | 0.42031 | differential | 13 | 128 | 512 | 39 | 0 | 0.1 | eq |
| 151 | 0.60818 | 0.56014 | 0.32715 | end-to-end | 1 | 64 | 256 | 9 | 0 | 0 | mid |
| 152 | 0.60822 | 0.71829 | 0.43185 | end-to-end | 9 | 64 | 512 | 29 | 0.1 | 0 | eq |
| 153 | 0.60836 | 0.59618 | 0.36764 | end-to-end | 1 | 256 | 1028 | 9 | 0.1 | 0.1 | eq |
| 154 | 0.60872 | 0.68428 | 0.41479 | differential | 13 | 128 | 256 | 29 | 0 | 0 | mid |
| 155 | 0.60916 | 0.56525 | 0.31619 | end-to-end | 5 | 256 | 1028 | 9 | 0 | 0.1 | eq |
| 156 | 0.60929 | 0.59259 | 0.34192 | end-to-end | 9 | 128 | 512 | 29 | 0.1 | 0.1 | eq |
| 157 | 0.60929 | 0.59259 | 0.34192 | end-to-end | 9 | 128 | 512 | 29 | 0.1 | 0.1 | eq |
| 158 | 0.60916 | 0.56525 | 0.31619 | end-to-end | 5 | 256 | 1028 | 9 | 0 | 0.1 | eq |
